# Supplementary material for: Uncertainty quantification techniques for data-driven space weather modeling: thermospheric density application
Source: Sci Rep. 2022 May 4;12:7256. doi: 10.1038/s41598-022-11049-3 (PMC9068719; doi:10.1038/s41598-022-11049-3)
Supplement: Supplementary file 1 — Supplementary Information. [file 41598_2022_11049_MOESM1_ESM.pdf]

Supplementary Material

Uncertainty Quantification Techniques for Space Weather Modeling: Thermospheric Density Application

Richard J. Licata, Piyush M. Mehta

Global CHAMP Maps as a Function of Altitude

To demonstrate global prediction of mean and standard deviation, the direct probability CHAMP model is evaluated at all EXTEMPLAR grid locations (for display purposes) for baseline conditions at seven altitudes. Supplementary Fig. S1 shows the global density maps and the associated uncertainties ($100 \cdot \sigma / \mu$). These maps show that the model makes sensible predictions at most altitudes. At 300 and 325 km, the model seems to predict a significant density maximum on the dayside that spans from 0800 to 2000 hours local time. Concurrently, the normalized uncertainty is largest in this region. Between 350 and 425 km, the predictions look as expected with an interesting trend of a shrinking region of high density on the dayside. The normalized uncertainties transition from most uncertain in the dayside to most uncertain in the night side. At 450 km, the peak shifts north and the uncertainties are on the order of 25\% of the mean prediction on the night side. This northern density shift is abnormal as these maps are all for the fall equinox. In general, the CHAMP model predicts normal density distributions between 350 and 425 km, where a majority of the training data resides. Relating these results to Fig. 9 in the manuscript, the global density maps may all look more physical if the solar activity used for each altitude were more representative of the solar activity encountered by CHAMP at each altitude.

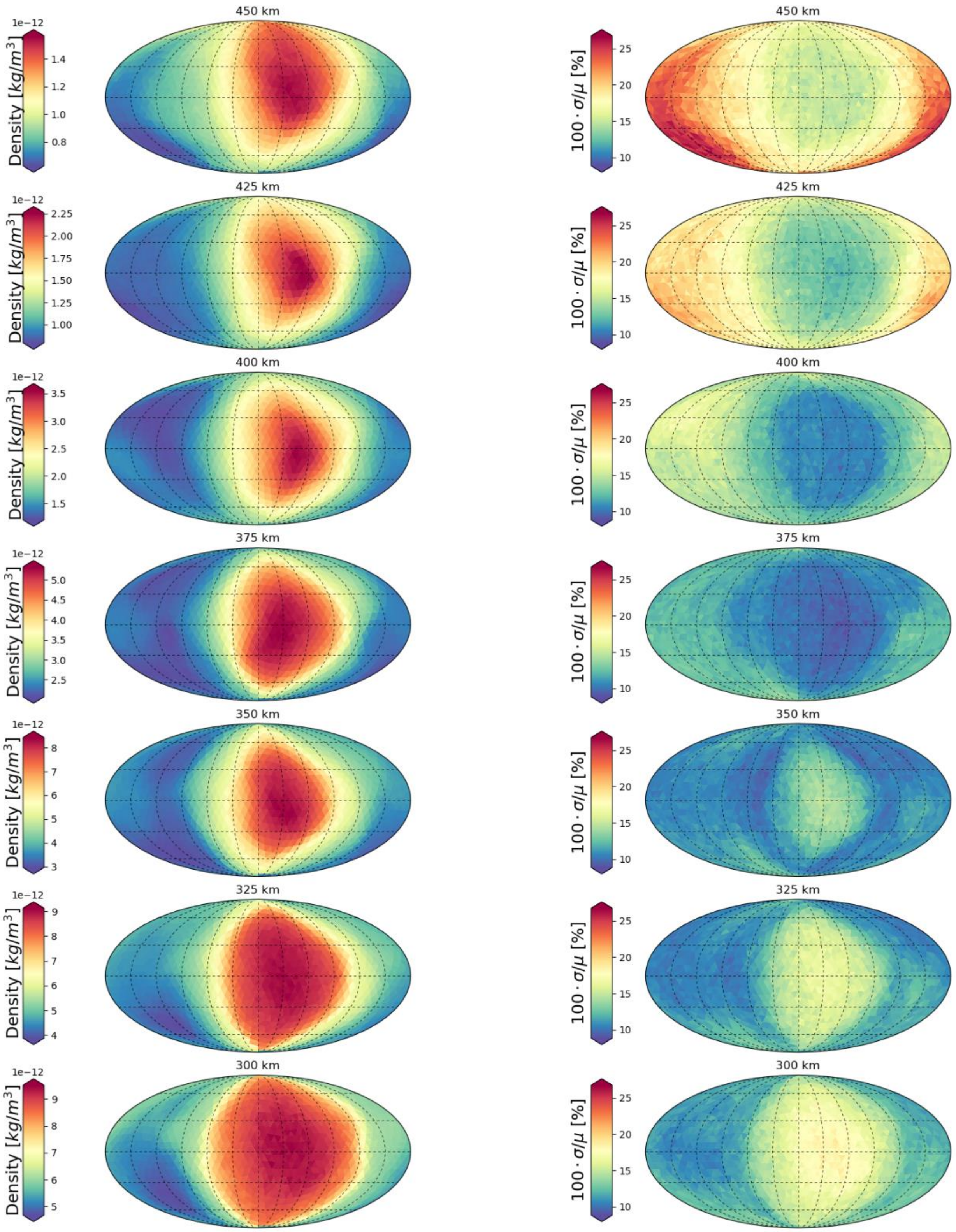

Supplementary Figure S1. Global density maps (left) for moderate solar and low geomagnetic activity with the associated uncertainties (right). Note: there are different colorbar limits for the density maps but a consistent colorbar is used for all uncertainty maps.

Storm Time Evaluation with Global Maps

We demonstrate global storm predictions by looking October 1, 2002 where *Dst* reached a minimum value of -176*nT*. The model inputs are averaged in 90 minute segments and an array of location inputs are provided for each of the 16 space weather conditions. The locations used are those used by the EXTEMPLAR model, but any locations can be input. The altitude was fixed at 412 km which was the average altitude of CHAMP during this period. We generated the global density maps (which represent the 90 minute average density distribution) and plot them with the mean absolute error along the CHAMP orbit. This is displayed in Fig. S2 and S3 showing the northern and southern hemispheres, respectively. Note: the direct probability model was used here for ease of evaluation as this is simply a demonstration of global prediction.

The maps in both figures show that even though the training data is local, the model is able to provide reasonable global representations of thermospheric mass density. The diurnal structure of the thermosphere is highlighted in the the first four segments, prior to the onset of the storm. The F_{10} and F_{81c} values for this day are 139.7 and 173.9 sfu, respectively. This indicates the thermosphere is highly driven by the temperature variations which is evident in both figures. The southern hemisphere (Fig. S3) displays a more significant impact of the storm as the high density values flow into the night-side at high latitudes. In the actual CHAMP data, the densities can often be erratic, particularly during storms, as a result of imperfect data processing schemes. However, these figures show that the model is able to learn the overall relationship of the inputs while being robust to outliers. This is emphasized by the continuity and smooth transitions between high- and low- density regions.

In terms of the model performance, the errors are lower in the northern hemisphere (16.84%) than in the southern hemisphere (17.19%). However, this could be a result of CHAMP's location during certain periods of the storm. The maximum errors (seen in dark red) are all in the southern hemisphere. There is also an interesting trend of higher errors in the night-side. This is because the density values are lowest here, so deviations from the true value have a stronger impact on the percent error. The error is shown as a percent for normalization as the density ranges by a factor of three as a function of location and time. It is important to reiterate that the density encountered by CHAMP at any point is not necessarily the density shown on the map as these are averages.

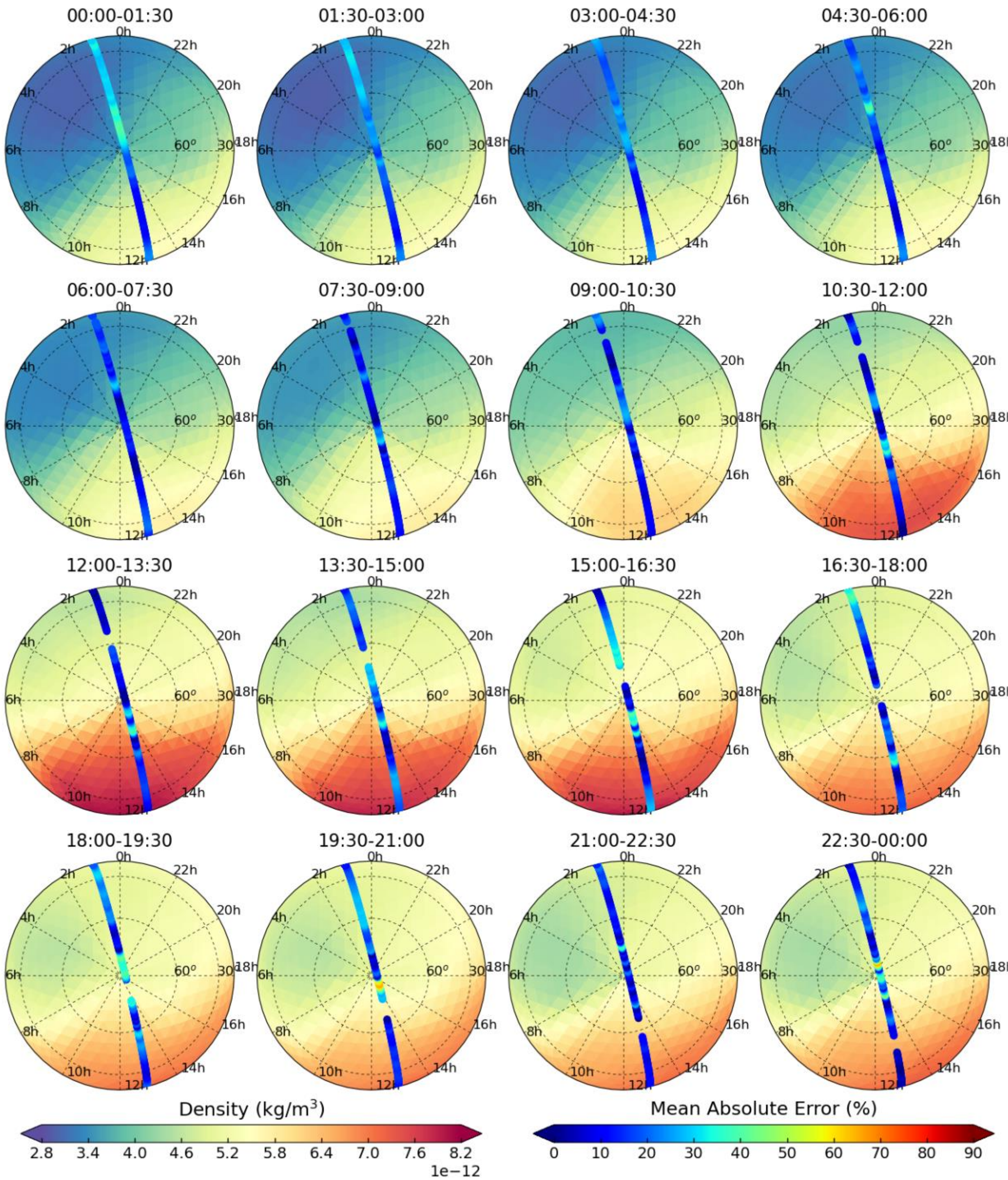

Supplementary Figure S2. 90 minute average density maps of the northern hemisphere on October 1, 2002. The line shows the flight path of CHAMP for that period with colors depicting the mean absolute error.

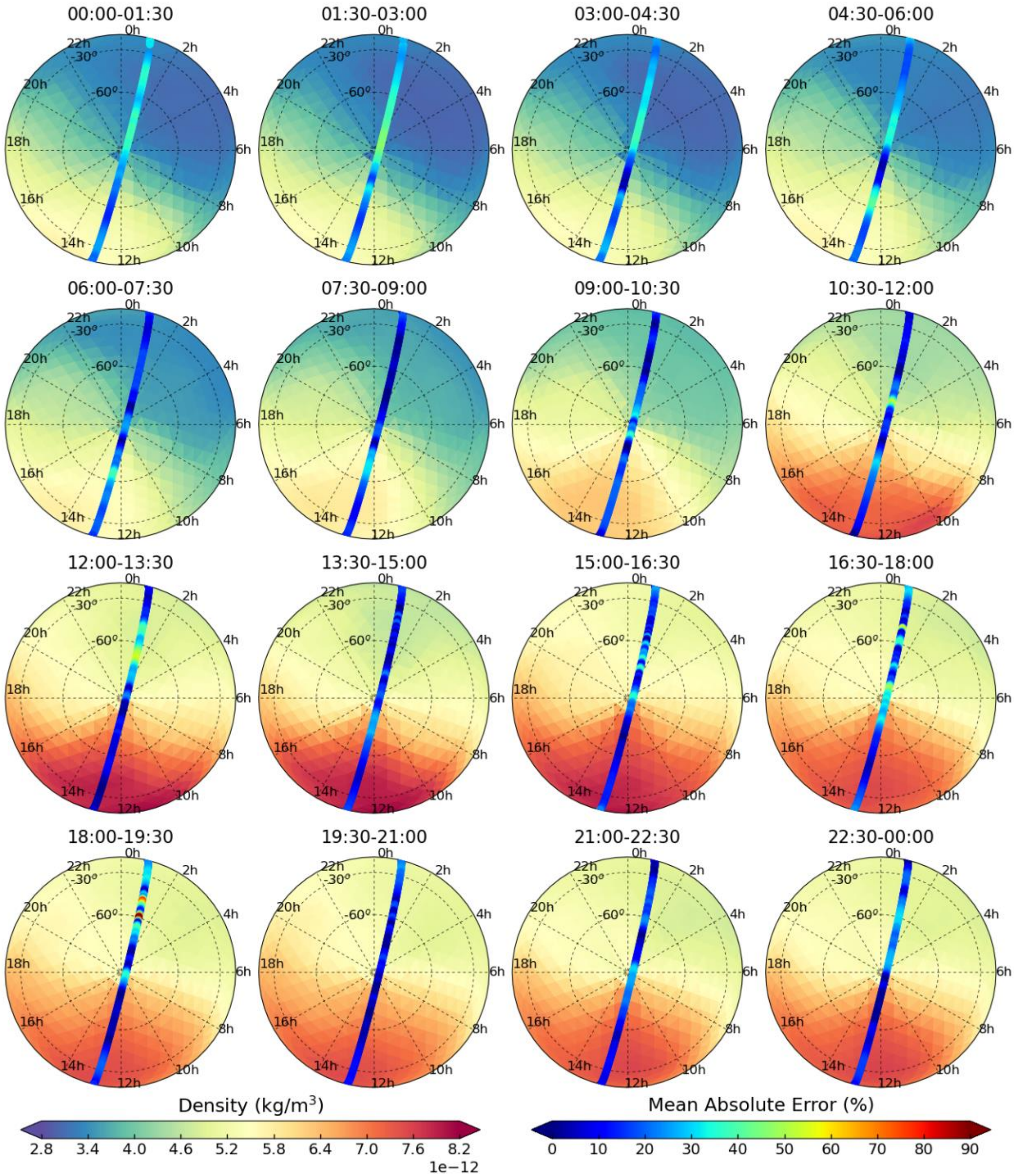

Supplementary Figure S3. 90 minute average density maps of the southern hemisphere on October 1, 2002. The line shows the flight path of CHAMP for that period with colors depicting the mean absolute error.
